# Exploring the Analgesic Initiation Mechanism of Tuina in the Dorsal Root Ganglion of Minor CCI Rats via the TRPV1/TRPA1-cGMP Pathway

**DOI:** 10.1155/2024/2437396

**Published:** 2024-07-24

**Authors:** Zhenjie Yang, Chula Sa, Tianyuan Yu, Jinping Chen, Runlong Zhang, Yingqi Zhang, Jiayue Liu, Hanyu Zhang, Jiawei Sun

**Affiliations:** School of Acupuncture-Moxibustion and Tuina Beijing University of Chinese Medicine, Beijing 102488, China

## Abstract

Tuina is a treatment method in traditional Chinese medicine which has analgesic effects and effectively alleviates the symptoms of neuropathic pain (NP). Transient receptor potential vanilloid type 1 (TRPV1) and transient receptor potential ankyrin type 1 (TRPA1) play major roles in transmitting nociceptive sensory signals in the nociceptive primary sensory dorsal root ganglion (DRG) nerve. The nitric oxide (NO)/cyclic guanosine 3′,5′-monophosphate(cGMP) pathway exerts both nociceptive and antinociceptive effects in various chronic pain models. TRPV1 and TRPA1 mediate the influx of calcium, which stimulates the generation of NO. Subsequently, NO activates the NO/cGMP/protein kinase G (PKG) signaling pathway, thereby improving hyperalgesia. In the present study, oa rat model of NP with minor chronic constriction injury (CCI) of the right sciatic nerve of NP was established. The results of behavioral testing showed that, after a one-time tuina intervention, the mechanical withdrawal threshold (MWT) and thermal withdrawal latency (TWL) were prolonged to varying degrees in the tuina group compared with the model group. Similarly, the expression of TRPV1, TRPA1, NO, soluble guanylate cyclase *β* (sGC*β*), cGMP, and PKG1 was significantly decreased in the DRG of the tuina and tuina + TRPV1/TRPA1 antagonist group was significantly decreased. These findings suggest that the tuina intervention can effectively improve the symptoms of thermal and mechanical allodynia caused by peripheral nerve injuries. Tuina exerts immediate analgesic effects through the TRPV1/TRPA1-NO-cGMP-PKG signaling pathway.

## 1. Introduction

Neuropathic pain (NP) refers to pain caused by a lesion or disease of the somatosensory nervous system. Its clinical manifestations mainly include spontaneous pain, hyperalgesia and allodynia, aftersensations, and referred pain [1, 2]. According to epidemiological analysis and statistics, nearly 5% of the world's population suffers from NP, with 18% in developing countries and 11–38% in adolescents, among whom 3–5% have severe disabilities [3–6]. According to the 11th revision of the International Statistical Classification of Diseases and Related Health Problems (ICD) published by the World Health Organization (WHO), the NP is divided into peripheral NP and central NP based on the position of the of the disease or lesion [7]. Currently, the main method for the clinical treatment of neuropathic pain is drug therapy, but this can only provide temporary pain relief for some patients; additionally, most drugs have side effects, such as dizziness, cardiotoxicity, erythema, addiction, and respiratory depression [8–13].

Tuina, an effective, green, and almost side-effect-free external treatment using traditional Chinese medicine, has significant immediate analgesic effects in the clinical treatment of many NP-related diseases [14–16]. The “Three-Manipulation and Three-Acupoint” method is employed based on the preliminary clinical experience and research results of the research group and essentially comprises an acupoint-nerve-muscle related area. The three manipulations are point-pressing, plucking, and kneading, which are warming and relaxing methods. The three acupoints, Yinmen (BL37), Chengshan (BL57), and Yanglingquan (GB34), are located on the femoral nerve and its branches [17]. In the early stage, the research group used transcriptome sequencing technology to analyze the spinal cord dorsal horn (SDH) and dorsal root ganglion (DRG) of minor chronic constriction injury (CCI) model rats following a one-time tuina intervention. The results demonstrated that 118 differentially expressed genes, including transient receptor potential (TRP) protein-related genes, showed significant changes and were enriched in 111 pathways including the NO-cGMP- nitric oxide (NO)/cyclic guanosine 3′,5′-monophosphate (cGMP)/protein kinase G (PKG) signaling pathway [18]. However, it is unknown how tuina exerts immediate analgesic effects through the TRP and NO/cGMP/PKG signaling pathways.

TRP vanilloid 1 (TRPV1) and TRP ankyrin 1 (TRPA1) increase neuronal activity and induce hypersensitivity following peripheral nerve damage [19–21]. TRPV1 is a cation channel which is activated by harmful heat stimuli and contributes to the progression of acute and chronic pain. Several pharmacological studies have shown that the expression of TRPV1 in the DRG of model rats increases after nerve damage and that the thermal hyperalgesia symptoms of NP model rats are significantly improved after the injection of TRPV1 antagonists [22–24]. In addition, multiple studies have shown that TRPA1 is a mechanosensitive cation channel, and inhibition of the expression of the TRPA1 can effectively alleviate mechanical nociception after peripheral nerve injury [25–27]. Approximately 30% of neurons express TRPV1 and TRPA1 together [28].

The NO/cGMP signaling pathway has nociceptive and antinociceptive effects in various models of chronic pain. NO is a gas, synthesized from arginine by neuronal nitric oxide synthase (nNOS), which is found in the superficial laminae (I–III) of the spinal cord and in DRG neurons [29, 30]. The calcium (Ca^2+^)-binding protein calmodulin (CaM) binds to Ca^2+^ and activates nNOS [31]. After peripheral nerve injury, the expression of TRPV1 and TRPA1 increases, and Ca^2+^ flow in and combine with CaM to act on nNOS to produce NO. As one of the subtypes of soluble guanylate cyclase (sGC), sGC*β* is mainly expressed in the SDH and DRG, which brings about subsequent production of cGMP after activation by NO [32]. PKG, which is a cGMP-dependent protein kinase, is then activated and is associated with the activation of multiple targets.

Minor CCI model rats were selected for the purpose of activating clinic NP. After a one-time intervention using “Three-Manipulation and Three-Acupoint” tuina, the analgesic effect was observed through behavioral and molecular biological experiments; we analyzed the proteins related to the TRPV1/TRPA1-NO-cGMP-PKG signaling pathway in the DRG, with a view to exploring whether tuina can initiate analgesia through this pathway.

## 2. Materials and Methods

### 2.1. Animals and Grouping

This study was approved by the Animal Ethics Committee of the Beijing University of Chinese Medicine (No. BUCM-4-2022082605-3043). During the feeding process, the 3R principle (replacement, reduction, and refinement) for the use of experimental animals was strictly followed, and humane care was provided daily by the experimental staff.

Male Sprague–Dawley (SD) rats aged 6–8 weeks and weighing 190–210 grams (g) were obtained from SPF Biotechnology Co., Ltd. (license No. SCXK (Jing) 2019-0010, Beijing, China). These rats were acclimated in the laboratory animal feeding room of the Beijing University of Chinese Medicine for seven days, with an indoor temperature of 25 ± 1°C and a humidity of 50 ± 5%. Fifty-six male SD rats were stochastically split into eight groups: the control group (CG), the sham group (SG), the model 1 group (M1), the model 2 group (M2), the tuina 1 group (T1), the tuina 2 group (T2), the tuina 1 + TRPV1 antagonist group (T1V1), and the tuina 2 + TRPA1 antagonist group (T2A1), with seven rats in each group.

### 2.2. Modeling

The rats were subjected to minor CCI modeling after seven days of adaptive feeding. The modeling method was based on previous descriptions and research conducted by research groups [33, 34]. Briefly, after the rats initially underwent inhaled anesthesia with 4%-5% isoflurane (RWD Life Science, Shenzhen, China), the isoflurane concentration was subsequently maintained at 3%. A 4-0 chromic absorbable surgical suture (Shandong Boda Medical Products, Shandong, China) was used to ligate the right sciatic nerve immediately before the bifurcation, and the force was adjusted to ensure that it did not affect blood flow outside the nerve. The nerve with no ligation was just exposed by the SG. In addition, the CG did not undergo surgery.

### 2.3. Intervention Methods

#### 2.3.1. Antagonist Injection

The rats in the T1V1 group were injected with the TRPV1 antagonist AMG517 (MedChemExpress, Shanghai, China) at a concentration of 0.3 mg/kg through the tail vein for three consecutive days after the modeling process [31]. The rats in the T2A1 antagonist group were injected with a TRPA1 antagonist A-967079 (MedChemExpress, Shanghai, China) at a concentration of 6.2 mg/kg through the tail vein, after the modeling process and 35 minutes (min) before the tuina intervention [35].

#### 2.3.2. Tuina

**Figure 1:**
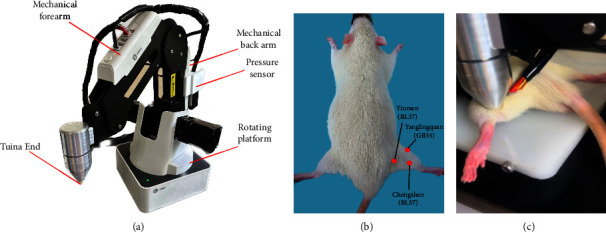
Intelligent tuina manipulation simulator treatment. (a) The intelligent tuina manipulation simulator (patent no. ZL ZL 2023 20511277.5). (b) The positions of Yinmen (BL37), Chengshan (BL57), and Yanglingquan (GB34). (c) Stimulating Yinmen (BL37).

Seven days after modeling, the T1, T 2, T1V1, and T2A1 groups were subjected to a one-time tuina intervention. A custom-made intelligent tuina manipulation simulator (intervention patent no. ZL202320511277.5, Beijing, China) (Figure 1(a)) was used to simulate the “Three-Manipulation and Three-Acupoint” treatment, and particularly, the point-pressing, plucking, and kneading manipulations of the right Yinmen (BL37), Chengshan (BL57), and Yanglingquan (GB34) (Figures 1(b) and 1(c)). Additionally, the parameters were set to a strength of 4 Newtons (N) and a frequency of 60 times/min. Each manipulation was applied to each acupoint for 1 min, for a total of 9 min. The rats in the SG, M1, and M2 groups were subjected to grasping restraints for 9 minutes each. The rats in the CG were not subjected to any interventions.

### 2.4. Behavioral Tests

The mechanical withdrawal threshold (MWT) and thermal withdrawal latency (TWL) of each group of rats were tested before modeling, preintervention, and postintervention. The CG, SG, M1, and T1 groups were tested for TWL immediately after the intervention. The CG, SG, M2, and T2 groups were tested for MWT 24 hours (h) after the intervention [36].

#### 2.4.1. TWL

A thermal pain instrument (PL-200, Chengdu Techman, China) was used to evaluate the TWL in each group of rats. The rats were placed in a test box with a perforated top and a transparent partition at the bottom, which was blackened to facilitate environmental adaptation. After 15–30 min of acclimatization, the exploratory activities of the rats ceased and the test was conducted. The instrument parameters were set to a maximum test time of 20 seconds (s) and an intensity of 50%. The infrared probe was moved to the center of the right foot of the rat, and the infrared switch was pressed to start the test. When the rat raised its foot and licked it, the latency of the foot withdrawal reflex was measured using the instrument. The measurements were performed five times in succession, with an interval of 10 min between each measurement.

#### 2.4.2. MWT

The MWT of each group of rats was evaluated using a Von Frey pain measurement instrument (BIO-EVF5, Bioseb, USA). The rats were placed into a transparent rat box with a baffle and a grid at the bottom and allowed to adapt to the environment for 15–30 min. When the exploratory of the rats' activities was ceased, the pain measurement instrument probe was moved to the center of the right foot of the rat, and the pressure was increased in a continuous and linear. The instrument display threshold was recorded when the rats raised and licked their feet. The measurements were conducted five times in succession, with an interval of 10 min between each measurement.

### 2.5. Enzyme-Linked Immunosorbent Assay (ELISA)

After all behavioral tests were completed in each group of rats, 1% sodium pentobarbital (Sigma-Aldrich LLC., Germany) was injected into the abdominal cavity for anesthesia and then fixed with 4% paraformaldehyde (Coolaber, Beijing, China). After the corneal reflex disappeared, the L_4-6_ DRG was removed and stored at -80°C.

After the tissue specimens were removed from the −80°C refrigerator, they were weighed and mixed with a certain amount of phosphate buffered saline (PBS; pH = 7.4) on ice. The homogenate was sonicated thoroughly and centrifuged at 3000 revolutions per minute (rpm) for 20 min. The supernatant was then collected for detection. The rat cGMP ELISA kit (Shanghai Enzyme-linked Biotechnology, Shanghai, China) was used according to its operating instructions to detect the expression level of cGMP in the L_4-6_ DRG of the rats in each group.

### 2.6. Nitrite Production Assay

When NO encounters oxygen and water, it generates nitrate and nitrite, which can then react with a nitrate developer to produce a reddish azo compound. The concentration of NO can indirectly be measured using colorimetry; an NO assay kit (Beyotime Biotech Inc., Jiangsu, China) based on this principle was used for measurements. After the DRG was removed from the −80°C refrigerator, it was weighed and mechanically homogenized according to the weight-to-volume ratio of physiological saline, which is 1 (g) : 9 (mL). The sample was then centrifuged at 3500 rpm for 10 min. After centrifugation, the supernatant, which is the 10% homogenized supernatant, was collected and divided into samples of 300 *μ*L each. The kit instructions were followed to detect the NO content in the L_4-6_ DRG of the rats in each group.

### 2.7. Western Blot

The DRG was added to a protein lysis solution (Servicebio Technology, Wuhan, China) and lysed on ice for 30 min. The sample was then centrifuged at 1200 rpm for 15 min before the supernatant was collected. A bicinchoninic acid (BCA) kit (Servicebio Technology, Wuhan, China) was used to determine the protein concentration in the supernatant. PBS and the loading buffer were added to configure the protein system based on the results of the concentration measurement. The protein loading amount was 30 *μ*g. According to the molecular weight, 6% and 10% SDS-PAGE gels were used to separate proteins, which were then transferred to a polyvinylidene fluoride (PVDF) membrane using the Sandwich transfer method. Fast sealing liquid (New Cell & Molecular Biotech, Suzhou, China) was used to slowly shake and seal the shaker for 30 min. The membranes were washed with phosphate buffered saline with Tween® 20 (PBST) for 10 min, and this process was repeated thrice; the primary antibodies were then incubated (Table 1) overnight at 4°C. The primary antibodies were collected and PBST was used to wash the membranes thrice, for 10 min each time. The corresponding secondary antibodies were added (Table 1) and the specimen was incubated at room temperature for 1 h on a shaking table. Electrochemical luminescence (ECL) was used to collect images on a universal imaging system, and the protein grayscale values were calculated using the Image J software.

### 2.8. Quantitative Reverse Transcription-Polymerase Chain Reaction

The total ribonucleic acids (RNAs) were extracted from the corresponding tissues and cells using the TRIzol reagent (Servicebio Technology, Wuhan, China). The RNA samples were reverse transcribed into complementary deoxyribonucleic acid (cDNA) using the SweScript All-in-One Reverse Transcription (RT) SuperMix for quantitative polymerase chain reaction (qPCR) (one-step genomic DNA (gDNA) remover) kit (Servicebio Technology, Wuhan, China), according to the instructions provided by the manufacturer. The amplification reaction was completed using a fluorescence qPCR instrument according to the instructions for the use of the 2 × Universal Blue SYBR Green qPCR Master Mix reagent. Primer sequences for glyceraldehyde 3-phosphate dehydrogenase (GAPDH), TRPV1, TRPA1, nNOS, sGC*β*, cGMP, and PKG1 are shown in Table 2.

### 2.9. Statistical Analysis

The results of the behavioral tests, ELISA, NO content detection, and qPCR were statistically analyzed using the SPSS software (version 26.0), and the grayscale values of the Western blot bands were analyzed using the Image J software. One-way analysis of variance (ANOVA) was performed to compare the results between groups, and differences were considered statistically significant when *P* < 0.05.

## 3. Results

### 3.1. Significant Improvements in TWL Immediately after Tuina

**Figure 2:**
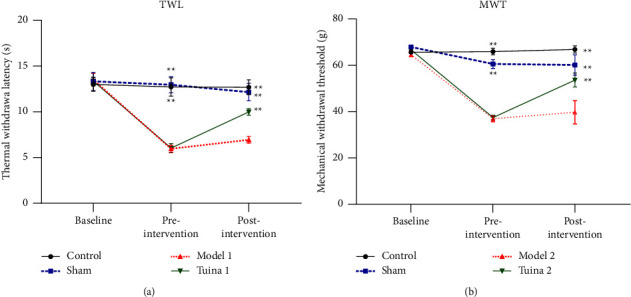
Results of behavioral tests in each group of rats ($\overset{¯}{x}$ ± *s*, *n* = 7). (a) Results of thermal withdrawal latency (TWL) in rats with thermal hyperalgesia. (b) Results of mechanical withdrawal threshold (MWT) in rats with mechanical allodynia. In comparison with the group model, ^*∗*^*P* < 0.05 and ^*∗∗*^*P* < 0.01.

As shown in Figure 2(a), no difference in baseline values was observed among the groups before modeling, indicating that the pain threshold was uniform. Before the “Three-Manipulation and Three-Acupoint” tuina intervention, the M1 and T1 groups showed statistically significant decreased in TWL compared with the CG and SG (*P* < 0.01). Immediately after the tuina intervention, the T1 exhibited a notable increase in TWL, and the difference was statistically significant (*P* < 0.01). Conversely, the M1 group showed no significant change in TWL, indicating that the “Three-Manipulation and Three-Acupoint” tuina intervention can effectively reduce thermal hyperalgesia and produce an immediate analgesic effect.

### 3.2. Significant Improvements in MWT 24 hours after Tuina

As shown in Figure 2(b), no difference in baseline values was observed among groups before modeling, indicating that the pain threshold was uniform. After modeling, the “Three-Manipulation and Three-Acupoint” tuina intervention was performed. In comparison with the CG and SG, the M2 and T2 groups showed statistically significant decreases in MWT (*P* < 0.01). After 24 h following the tuina intervention, compared to M2, the T2 group showed a statistically significant increase in MWT compared with the M2 group (*P* < 0.01). In contrast, the M2 group showed no prominent change in MWT, indicating that the “Three-Manipulation and Three-Acupoint” tuina intervention can effectively alleviate mechanical allodynia.

### 3.3. Changes in cGMP Expression Levels in the L_4-6_ DRG of the Rats in Each Group

**Figure 3:**
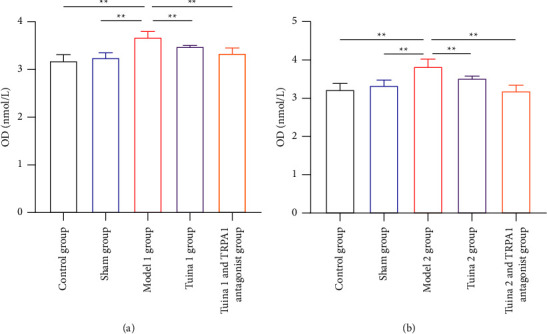
Results of cyclic guanosine 3′,5′-monophosphate (cGMP) protein content in the dorsal root ganglion (DRG) of rats in each group ($\overset{¯}{x}$ ± *s*, *n* = 7). (a) Results of cGMP protein content in the DRG of rats with thermal hyperalgesia. (b) Results of cGMP protein content in the DRG of rats with mechanical allodynia. In comparison with the group model, ^*∗*^*P* < 0.05 and ^*∗∗*^*P* < 0.01.

As shown in Figure 3(a), in the group with thermal hyperalgesia, cGMP expression in the M1 group significantly increased, compared with that in the CG and SG after the tuina intervention and intravenous injection of the TRPV1 antagonist (*P* < 0.01). Compared with the M1 group, the T1 and T1V1 groups showed significant decrease in cGMP expression (*P* < 0.01).

As shown in Figure 3(b), in the group with mechanical allodynia, cGMP expression in the M2 group significantly increased in comparison with that in the CG and SG after the tuina intervention and intravenous injection of the TRPA1 antagonist (*P* < 0.01). Compared with the M2 group, the T2 and T2A1 groups showed significant decreases in cGMP expression (*P* < 0.01).

In summary, the “Three-Manipulation and Three-Acupoint” tuina intervention can effectively reduce cGMP expression in the DRG of minor CCI model rats.

### 3.4. Changes in NO Content in the L_4-6_ DRG of the Rats in Each Group

**Figure 4:**
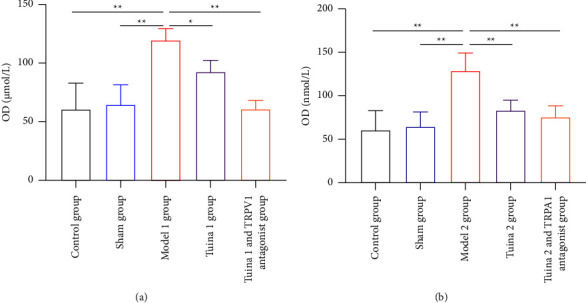
Results of nitric oxide (NO) content in the dorsal root ganglion (DRG) of the rats in each group ($\overset{¯}{x}$ ± *s*, *n* = 7). (a) Results of NO content in the DRG of rats with thermal hyperalgesia. (b) Results of NO content in the DRG of rats with mechanical allodynia. In comparison with the group model, ^*∗*^*P* < 0.05 and ^*∗∗*^*P* < 0.01.

As shown in Figure 4(a), in the group with thermal hyperalgesia, the NO content in the M1 group significantly increased in comparison with CG and SG after the tuina intervention and intravenous injection of the TRPV1 antagonist (*P* < 0.01); Compared with the M1 group, the T1 and T1V1 groups showed significant decreases in NO content (*P* < 0.05 and *P* < 0.01, respectively).

As shown in Figure 4(b), in the group with mechanical allodynia, the NO content in the M2 group significantly increased in comparison with that in the CG and SG after the tuina intervention and tail vein injection of the TRPA1 antagonist (*P* < 0.01).

In summary, the “Three-Manipulation and Three-Acupoint” tuina intervention can effectively reduce NO expression in the DRG of the minor CCI model.

### 3.5. Changes in the Expression of TRPV1, TRPA1, nNOS, sGC*β*, and PKG1 Proteins in the L_4-6_ DRG of the Rats in Each Group

**Figure 5:**
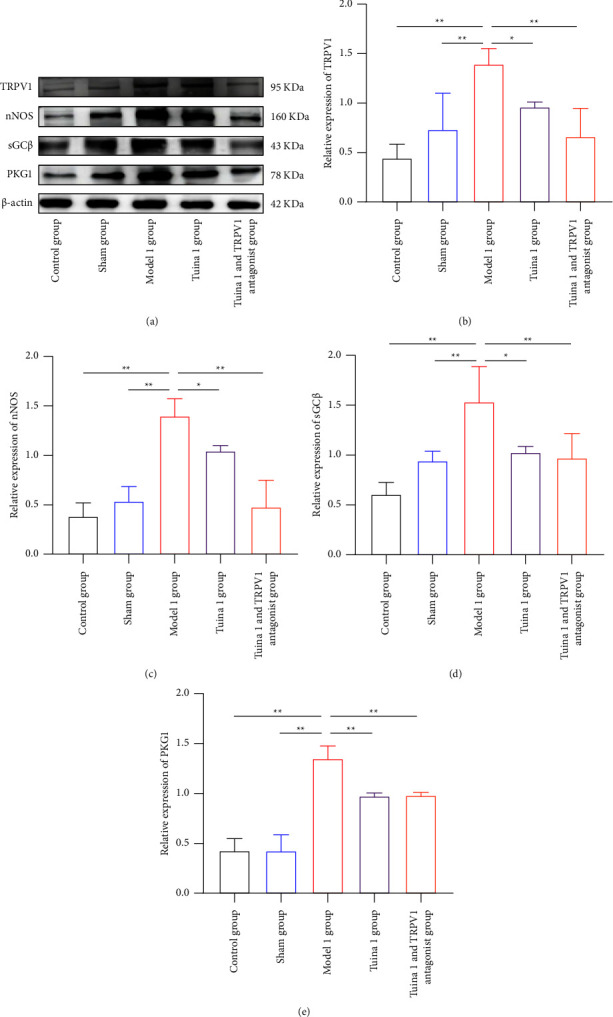
Effect of tuina on the expression of TRPV1/cGMP signaling pathway-related proteins in the DRG of minor CCI rats. (a)–(e) Representative gels and quantification of bands for TRPV1, nNOS, sGC*β*, and PKG1 in the DRG of rats with thermal hyperalgesia ($\overset{¯}{x}$ ± *s*, *n* = 3). In comparison with the group model, ^*∗*^*P* < 0.05 and ^*∗∗*^*P* < 0.01.

As shown in Figure 5, in the group with thermal hyperalgesia, the level of the four proteins (TRPV1, nNOS, sGC*β*, and PKG1) in the M1 group was significantly increased compared with that in the CG and SG, after the tuina intervention and tail vein injection of the TRPV1 antagonist (*P* < 0.01) (Figures 5(a), 5(b), 5(c), 5(d), and 5(e)). Compared with the M1 group, the T1 group showed significantly decreased levels of TRPV1, nNOS, and sGC*β* (*P* < 0.05) (Figures 5(a), 5(b), 5(c), and 5(d)), as well as significantly decreased levels of PKG1 (*P* < 0.01) (Figures 5(a) and 5(e)). Compared with the M1 group, the T1V1 group also showed significantly decreased levels of all four proteins (*P* < 0.01) (Figures 5(a), 5(b), 5(c), 5(d), and 5(e)).

**Figure 6:**
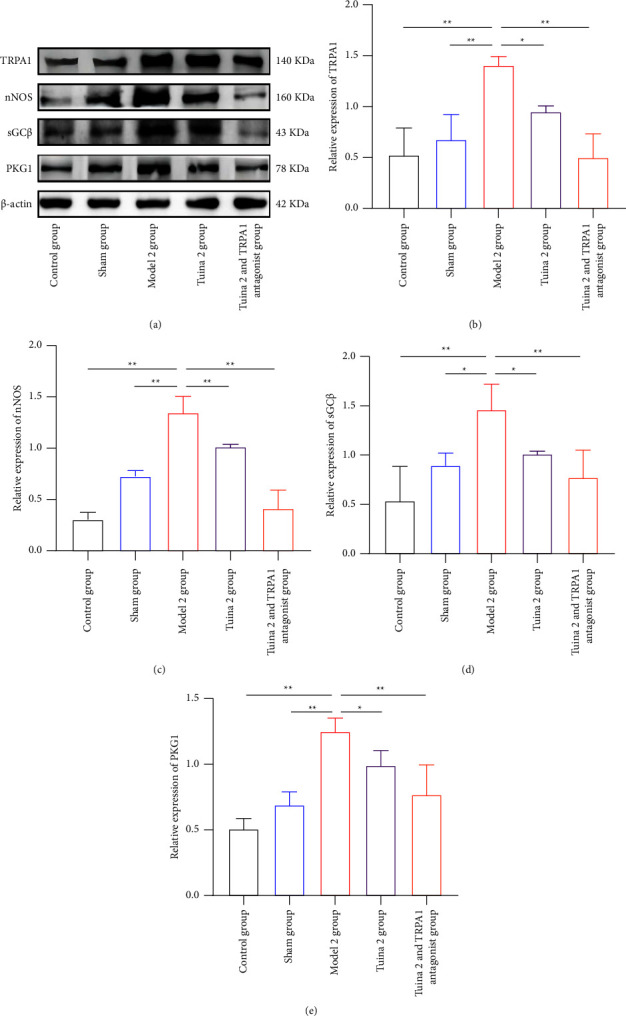
Effect of tuina on the expression of TRPA1/cGMP signaling pathway-related proteins in the DRG of minor CCI rats. (a)–(e) Representative gels and quantification of bands for substance TRPA1, nNOS, sGC*β*, and PKG1 in the DRG of rats with the mechanical allodynia group ($\overset{¯}{x}$ ± *s*, *n* = 3). Compared with the group model, ^*∗*^*P* < 0.05 and ^*∗∗*^*P* < 0.01.

As shown in Figure 6, in the group with mechanical allodynia, the levels of four proteins (TRPA1, nNOS, sGC*β*, and PKG1) in the M2 group were significantly increased compared with those in the CG and SG, after the tuina intervention and intravenous injection of the TRPA1 antagonist (*P* < 0.01, *P* < 0.05) (Figures 6(a), 6(b), 6(c), 6(d), and 6(e)). Compared with the M2 group, the T2 group showed significantly decreased levels of TRPA1, sGC*β*, and PKG1 (*P* < 0.01) (Figures 6(a), 6(b), 6(d), and 6(e)), as well as significantly decreased levels of nNOS (*P* < 0.05) (Figures 6(a) and 6(c)). Compared with the M2 group, the T2A1 group also showed significantly decreased levels of all four proteins (*P* < 0.01) (Figures 6(a), 6(b), 6(c), 6(d), and 6(e)).

In summary, the “Three-Manipulation and Three-Acupoint” tuina intervention can effectively reduce the expression of TRPV1, TRPA1, nNOS, sGC*β*, and PKG1 proteins in the DRG of minor CCI model rats.

### 3.6. Changes in the Expression of TRPV1, TRPA1, nNOS, sGC*β*, and PKG1 Genes in the L_4-6_ DRG of the Rats in Each Group

**Figure 7:**
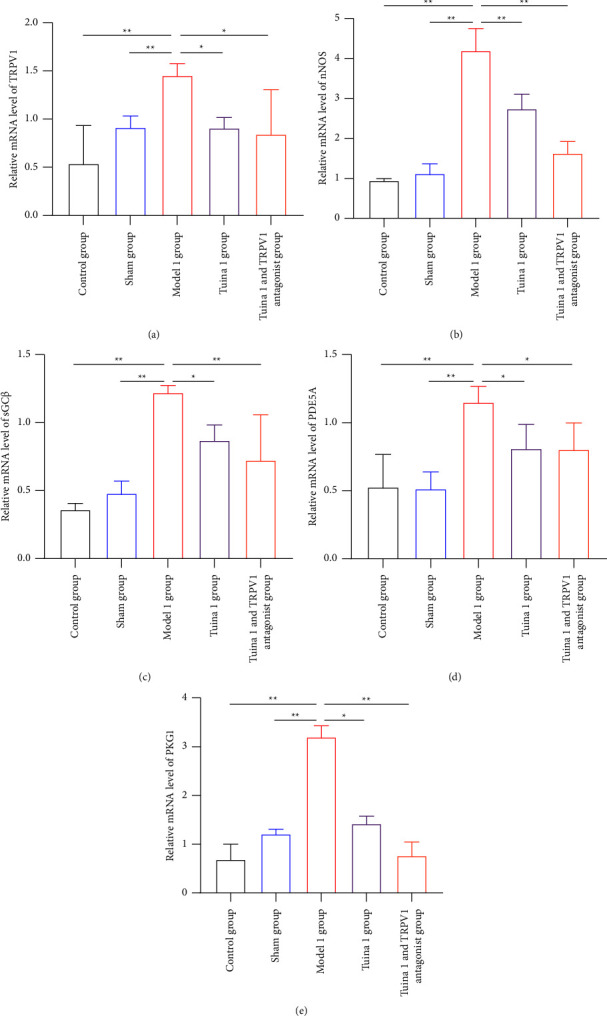
Effect of tuina on the expression of TRPV1/cGMP signaling pathway-related genes in the DRG of minor CCI rats. (a)–(e) Quantitative RT-PCR showing the downregulated mRNA levels of TRPV1, nNOS, sGC*β*, PDE5A(cGMP), and PKG1 in the DRG of minor CCI rats with thermal hyperalgesia ($\overset{¯}{x}$ ± *s*, *n* = 3). In comparison with the group model, ^*∗*^*P* < 0.05 and ^*∗∗*^*P* < 0.01.

As shown in Figure 7, in the group with thermal hyperalgesia, the expression levels of the five protein genes (TRPV1, PDE5A (cGMP), nNOS, sGC*β*, and PKG1) in the M1 group were significantly increased compared with those in the CG and SG after the tuina intervention and tail vein injection of the TRPV1 antagonist (*P* < 0.01) (Figures 7(a), 7(b), 7(c), 7(d), and 7(e)). Compared with the M1 group, the T1 group showed significantly decreased expression levels of the TRPV1, sGC*β*, PDE5A (cGMP), and PKG1 genes (*P* < 0.05) (Figures 7(a), 7(c), 7(d), and 7(e)), as well as significantly decreased expression levels of the nNOS gene (*P* < 0.01) (Figure 7(b)). Compared with the M1 group, the T1V1 group also showed significantly decreased expression levels of the TRPV1 and PDE5A (cGMP) genes (*P* < 0.05) (Figures 7(a) and 7(d)), as well as significantly decreased expression levels of the PKG1, nNOS, and sGC*β* genes (*P* < 0.01) (Figures 7(b), 7(c), and 7(e)).

**Figure 8:**
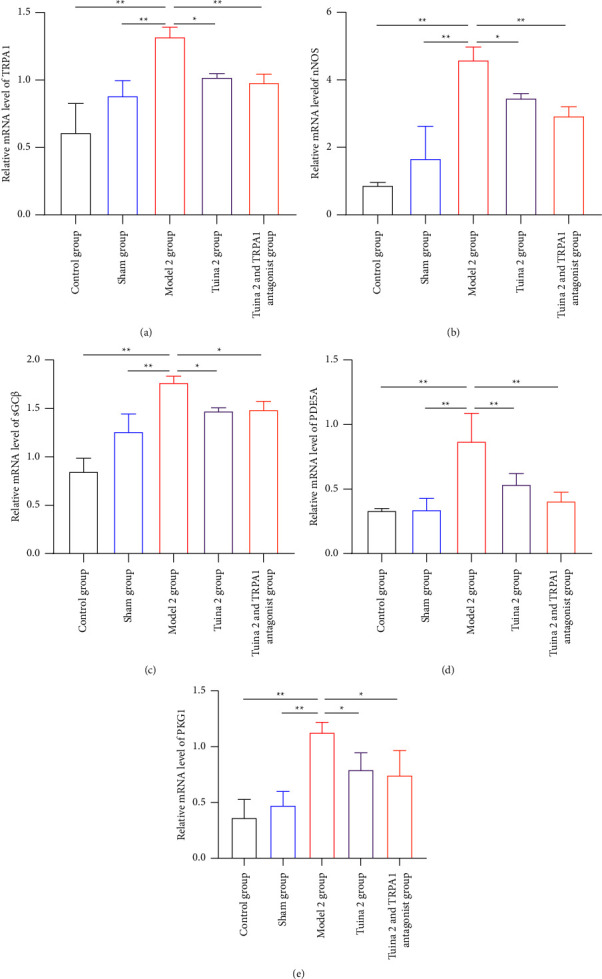
Effect of tuina on the expression of TRPA1/cGMP signaling pathway-related genes in the DRG of minor CCI rats. (a)–(e) Quantitative RT-PCR showing the downregulated mRNA levels of TRPA1, nNOS, sGC*β*, PDE5A(cGMP), and PKG1 in the DRG of minor CCI rats with mechanical allodynia ($\overset{¯}{x}$ ± *s*, *n* = 3). In comparison with the group model, ^*∗*^*P* < 0.05 and ^*∗∗*^*P* < 0.01.

As shown in Figure 8, in the group with mechanical allodynia, the expression levels of the five protein genes (TRPA1, PDE5A (cGMP), nNOS, sGC*β*, and PKG1) in the M2 group were significantly increased compared with those in the CG and SG after tuina intervention and tail vein injection of TRPA1 antagonist (*P* < 0.01) (Figures 8(a), 8(b), 8(c), 8(d), and 8(e)). Compared with the M2 group, the T2 group showed significantly decreased expression levels of the TRPA1, nNOS, sGC*β*, and PKG1 genes (*P* < 0.05) (Figures 8(a), 8(b), 8(c), and 8(e)) as well as significantly decreased expression levels of the PDE5A (cGMP) gene (*P* < 0.01) (Figure 8(d)). Compared with the M2 group, the T2A1 group also showed significantly decreased expression levels of the TRPA1, nNOS, PDE5A (cGMP), and PKG1 genes (*P* < 0.01) (Figures 8(a), 8(b), 8(d), and 8(e)) as well as significantly decreased expression levels of the sGC*β* gene (*P* < 0.05) (Figures 8(c)).

In summary, the “Three-Manipulation and Three-Acupoint” tuina intervention can effectively reduce the expression of TRPV1, TRPA1, nNOS, PDE5A (cGMP), PKG1, and sGC*β* gene in the DRG of minor CCI model rats.

## 4. Discussion

TRPV1 and TRPA1 are typical ion channel proteins in the TRP family that sense harmful thermal and mechanical stimuli and are mainly expressed in the DRG and other primary sensory neuron subsets. TRPV1 is a voltage-gated Ca^2+^ ion channel that senses harmful thermal stimuli, and its expression is increased in several neuropathic pain models, such as lumbar DRGs and unharmed spared nerve after spinal nerve ligation, partial nerve damage, or CCI [37–40]. This upregulation is associated with the progression and maintenance of thermal hyperalgesia in the corresponding hind paw. RPV1 receptor antagonists have been shown to effectively reduce the expression of TRPV1 in the DRG of rats with sciatic nerve damage and to improve the corresponding behaviors [41]. In neurogenic pain caused by nerve damage, the progression of mechanical allodynia is largely dependent on changes in the TRPA1 channel [42, 43]. Research has investigated the function of TRPA1 detecting mechanical stimuli in primary cells such as sensory nerve, odontoblasts, Merkel cells, and human periodontal ligament cells [44–47]. Furthermore, studies have found that TRPA1 antagonist can effectively reduce mechanically induced C fiber action potentials in wild-type mice and rats, especially under high-intensity forces [44].

Tuina can improve the symptoms of hyperalgesia in cases of NP by reducing the expression of TRPV1 and TRPA1 in the DRG of minor CCI model rats. In previous studies, tuina demonstrated an analgesic role and was capable of effectively reducing the expression of TRPA1 in the DRG of sciatic nerve damage model rats after 20 days s of tuina intervention, which plays an analgesic role [48]. Moreover, changes in the expression of related TRP protein genes were found in gene sequencing studies of one-time tuina interventions [18]. In this study, TWL and MWT were used as behavioral detection methods to detect the changes in thermal and mechanical hypersensitivity, in minor CCI model rats. Both thermal pain sensation and mechanical hypersensitivity significantly improved after tuina, with the former showing immediate improvement and the latter improving 24 h after the intervention; this result once again verified the analgesic effect of a one-time tuina intervention. Subsequently, the DRG of each group was removed to detect the expression levels of TRPV1 and TRPA1 protein and genes. The expression levels of proteins in the tuina and tuina + antagonist groups were significantly decreased, indicating that the “Three-Manipulation and Three-Acupoint” tuina intervention can effectively reduce the expression of TRPA1 and TRPV1 in the DRG of minor CCI model rats, thereby improving their pain hypersensitivity symptoms, and exerting an instant analgesic effect.

Tuina can initiate analgesia through the TRPV1/TRPA1-NO-cGMP-PKG signaling pathway. When the body is subjected to noxious stimuli, the TRP channels in primary sensory neurons in the DRG open; this promotes the influx of calcium, which couples with CaM, and acts on nNOS to produce NO using molecular oxygen and L-arginine as substrates [49, 50]. NO mostly acts on adjacent cells or cells where it is located by diffusion; it binds to the heme module of cytosolic sGC, catalyzes the transformation of guanosine triphosphate (GTP) into CGMP, and increases cGMP levels, thereby further activating cGMP-dependent PKG and promoting the release of pain mediators [51]. The nNOS is a pathological and physiological molecule involved in pain regulation in peripheral nerve tissues, whereas PKG1, one of the two subtypes of PKG, is widely distributed [52]. In this study, AMG517 and A-967079 were chosen as the respective TRPV1 and TRPA1 receptor antagonists. Furthermore, ELISA, Western blot, qPCR, and other methods were used to investigate changes in related proteins in the downstream NO-cGMP-PKG signaling pathway. The NO content and the expression of nNOS, cGMP, sGC*β*, and PKG1 in both the tuina and the tuina + antagonist groups decreased significantly, indicating that tuina can activate an analgesic mechanism by reducing the expression of related protein in the TRPV1/TRPA1-NO-cGMP-PKG signaling pathway, thereby exerting analgesic effects.

## 5. Conclusions

In summary, minor CCI model rats were studied with the aim of simulating clinical NP. The analgesic effect of tuina was verified after one session using various behavioral and molecular biological methods. The study also showed that tuina can activate an the analgesic mechanism by regulating the expression of related proteins in the TRPV1/TRPA1-NO-cGMP-PKG signaling pathway, thereby alleviating the symptoms of thermal and mechanical allodynia caused by peripheral damage.

## Figures and Tables

**Table 1:** Primary and secondary antibodies used for Western blotting.

Category	Antibodies	Concentration	Source
Primary	Mouse anti-TRPV1 monoclonal IgG	1 : 1000	Proteintech Group, Chicago, USA
Primary	Rabbit anti-TRPA1 polyclonal IgG	1 : 1000	Proteintech Group, Chicago, USA
Primary	Rabbit anti-nNOS polyclonal IgG	1 : 1000	Proteintech Group, Chicago, USA
Primary	Rabbit anti-sGC*β* polyclonal IgG	1 : 1000	Proteintech Group, Chicago, USA
Primary	Rabbit anti-PKG1 polyclonal IgG	1 : 1000	Proteintech Group, Chicago, USA
Primary	Rabbit anti-*β*-actin polyclonal IgG	1 : 1000	Proteintech Group, Chicago, USA
Secondary	Goat anti-rabbit IgG-HRP	1 : 6000	Zhong Shan-Golden Bridge Biological Technology, Beijing, China
Secondary	Goat anti-mouse IgG-HRP	1 : 6000	Zhong Shan-Golden Bridge Biological Technology, Beijing, China

**Table 2:** Sequences of the oligonucleotide primers.

Gene	Primer sequences (5′-3′)	Product size (bp)
GAPDH	CTGGAGAAACCTGCCAAGTATG GGTGGAAGAATGGGAGTTGCT	138

TRPV1	ACGACTTCAAGGCTGTCTTCATC TAGTCCAGTTTACCTCGTCCACC	294

TRPA1	TCTTCCTGCTATTGGCTTTTGG ATCTCGGTAATTGATGTCTCCCA	128

nNOS	GCCAAAGCAGAGATGAAAGACAC CCCAGTTCTTGACCTTGAGGAA	250

sGC*β*	CCTCAATGGCACTGTGATGGT ATGCGTGTTTCCACAAGGGTT	187

PDE5A (cGMP)	AAGCAGGCAAGATTCAGAACAAG CTGGGCTGTTTAGAACCATCAA	201

PKG1	GGTCACTGGTGTATGTCATGGAA TCCGGGTACAGTTGTAAAGAATAGC	123

## Data Availability

All the data information is included within the manuscript. If a data table is necessary, it will be provided as supplementary data.
